# 

**Published:** 1801-07

**Authors:** 


### MEDICAL AND PHYSICAL JOURNAL,

CONTAINING

*THE EARLIEST INFORMATION*

ON SUBJECTS OF

MEDICINE, SURGERY, PHARMACY, CHEMISTRY,

AND NATURAL HISTORY;

AND

*A critical Analysis of all new Works*

IN THOSE DEPARTMENTS OF LITERATURE.

CONDUCTED BY

T. BRADLEY, M. D.

Member of the Royal College of Physicians; Physician to the Westminster Hospital, and to the Asylum for Female Orphans; Lecturer on the Theory and Practice of Medicine, &c.

BY

R. BATTY, M. D.

Licentiate in Midwifery, of the Royal College of Physicians; Physician to the British Lying-in-Hospital; and to the Infant Asylum; Fellow of the Linnean Society; and Lecturer on Midwifery.

AND BY

A. A. NOEHDEN, M.D.

Vice-Secretary of the Physical Society of Gottingen; Corresponding Member of the Linnean Society, London, and of the Medical Society at Paris.

VOL. VI.

FROM JULY TO DECEMBER, 1801.

LONDON:

Printed for R. PHILLIPS, No. 71, ST. PAUL'S CHURCH-YARD; and sold by all Booksellers in the United Kingdoms.

*Price 15s. 6d. in Boards.*

Gillet, Salisbury Square.

### To CORRESPONDENTS.

*Receiving Dr. Jenner's Account of the Origin of Vaccine Inoculation, with the words* "From the Author," *we had no doubt that it was* published; *we now understand that Dr. J. printed but a small number to distribute among his friends.*

